# HIV/AIDS knowledge and attitude towards people living with HIV/AIDS (PLWHA): a cross-sectional study of primary school teachers

**DOI:** 10.4314/ahs.v20i4.11

**Published:** 2020-12

**Authors:** Ignatius O Nwimo, Nwamaka A Elom, Cajetan I Ilo, Rita N Ojide, Uchechukwu A Ezugwu, Vitalis U Eke, Lazaus E Ezugwu

**Affiliations:** 1 Department of Human Kinetics and Health Education, Ebonyi State University, Abakaliki, Nigeria; 2 Department of Medical Rehabilitation, University of Nigeria, Nsukka, Nigeria; 3 Department of Vocational and Special Education, University of Calabar, Calabar, Nigeria; 4 College of Health Technology, Oji-River, Enugu State, Nigeria

**Keywords:** HIV/AIDS, knowledge, attitude, PLWHA, primary school, teachers

## Abstract

**Background:**

Teachers are in advantage position to propagate correct information with regard to HIV/AIDS thereby influencing attitude towards PLWHA. With correct information stigmatization leading to spread of the scourge might be prevented.

**Aims & Objectives:**

The study was conducted to determine knowledge and attitude of primary school teachers towards PLWHA.

**Methods & Materials:**

The cross-sectional survey was used to study a sample of 400 primary school teachers in Ebonyi State, Nigeria. The instrument used for data collection was researchers' designed questionnaire. Out of 400 copies of questionnaire administered; 394 representing 98.5% return rate, were used for analysis of data.

**Results:**

Results showed respondents had moderate (57.4%) knowledge concerning HIV/AIDS and positive attitude (3.09 ± 0.98) to PLWHA. Female teachers' dispositions to PLWHA were better than the males based on their attitude scores and the difference was not significant in general knowledge of HIV/AIDS and attitude to PLWHA.

**Conclusion:**

Our findings underscore the need for a universal health education programme, focusing on HIV/AIDS education, in education institutions that train teachers in Nigeria so as to possibly mitigate the discrepancy in knowledge regarding curability of AIDS and any undesirable attitude towards PLWHA that may arise among teachers.

## Introduction

The Joint United Nations Programme on HIV/AIDS (UNAIDS) noted that human immunodeficiency virus (HIV) is the causative organism of acquired immunodeficiency syndrome (AIDS). It was first reported in San Francisco, USA, in previously well individuals with impaired immune function as evidenced by depletion of CD4 T helper lymphocyte. HIV/AIDS has emerged as the most significant infectious pathogen that is advancing relentlessly into the current century^1^. In countries the world over, HIV/AIDS is manifesting as an epidemic of unprecedented magnitude^2^, ^3^. A superfluity of epidemiological indicators point to the current and rising impact of the epidemic on individuals, families and societies^4^. Global aggregates of HIV/AIDS occurrence show that over 60 million people have lived with HIV/AIDS since the beginning of the epidemic; about 20 million of these have already died from AIDS^5^.

When disaggregated geographically, the HIV/AIDS pandemic shows widespread but uneven distribution in countries world-wide. Countries of sub-Saharan Africa are known to be most severely affected being host to 26.6 million infections. The recent UNAIDS report aptly points out that ‘Southern Africa is home of about 30% of people living with HIV/AIDS world-wide’ even though it represents less than 2% of the world's population^4^.

The impact of HIV and AIDS permeates all boundaries and has kept the economies of countries in sub-Saharan Africa in a perilous state. The extent of the problem of HIV and AIDS in the region indicates that Zambia, Namibia, South Africa, Lesotho, Zimbabwe, Swazilnd and Botswana are hardest hit with infection rates of 20.0%, 20.0%, 22.6%, 23.6%, 25.1%, 25.1% and 35.8% respectively. Data from the Democratic Republic of Congo (DRC) and Angola are regarded as unreliable, due to the effect of war in undertaking authentic HIV and AIDS surveillance^6^–^8^. Human Development Index (HDI) ranking which is an index of quality of life, life expectancy and population growth rates have markedly declined over the last 5 to 15 years^9^. Sub-Saharan Africa remains the region most heavily affected by HIV. In 2008, sub-Saharan Africa accounted for 67% of HIV infections worldwide, 68% of new HIV infections among adults and 91% of new HIV infections among children. The region also accounted for 72% of the world's AIDS-related deaths in 2008^10^.

The epidemic continues to have an enormous impact on households, communities, businesses, public services and national economies in the region. In Swaziland, Nigeria and most other countries in sub-Saharan Africa average life expectancy fell by half between 1990 and 2007, to 37 and 38.3 years respectively^11^–^13^. In 2008, more than 14.1 million (11.5 million–17.1 million) children in sub-Saharan Africa were estimated to have lost one or both parents to AIDS.

Women and girls continue to be affected disproportionately by HIV in sub-Saharan Africa. For example, in Côte d'Ivoire, home to the most serious epidemic in West Africa, HIV prevalence among females (6.4%) was more than twice as high as among males (2.9%) in 2005. In sub-Saharan Africa as a whole, women account for approximately 60% of estimated HIV infections^14^–^16^. As the epidemic has evolved in sub-Saharan Africa, the relationship between HIV infection and education has shifted, according to a recent meta-analysis of 36 studies carried out in 11 countries between 1987 and 2003. While studies prior to 1996 generally found either no association between educational status and HIV risk or found that the highest risk was among the most educated, data collected after 1996 have tended to find a lower risk among the most-educated people^17^.

HIV prevalence tends to be higher in urban settings than in rural areas, with household surveys conducted in the region between 2001 and 2005 indicating that the median urban/rural ratio of HIV prevalence is 1.7:1.015. In the sub-Saharan African countries where household surveys have been conducted, HIV prevalence is higher in rural areas only in Senegal. The most pronounced difference in HIV prevalence is in Ethiopia, where urban dwellers are eight times more likely to be HIV-infected than people living in rural areas^19^.

Although HIV prevalence in West and Central Africa is much lower than in Southern Africa, the sub-region nevertheless is home to several serious national epidemics. While adult HIV prevalence is below 1% in three West African countries (Cape Verde, Niger and Senegal), nearly one in 25 adults (3.9%) in Côte d'Ivoire and 1.9% of the general population in Ghana are living with HIV^16^. A 2007 household survey found that HIV prevalence in the Democratic Republic of the Congo (1.3%) remains significantly lower than in several other neighbouring countries^20^.

Some further favourable signs are apparent in the sub-region. Multiple household surveys have detected declining HIV prevalence in Mali (from 1.7% in 2001 to 1.2% in 2006) and Niger (from 0.9% in 2002 to 0.7% in 2006). In Benin, the percentage of antenatal clinic attendees who tested HIV-positive fell by almost half between 2001 and 2007, from 4.1% to 2.1%^21^. Declines in HIV prevalence among antenatal clinic attendees have also been documented in Burkina Faso, Côte d'Ivoire and Togo. While HIV prevalence in the general population in Ghana has remained stable, prevalence among 15–24-year-olds fell from 3.2% in 2002 to 2.5% in 2006. Other national epidemics also appear to have stabilized, including in Sierra Leone, where population-based surveys in 2005 and 2008 both found an overall HIV prevalence of 1.5%^22^.

The first case of HIV/AIDS was identified in Nigeria in 1986 and the HIV/AIDS prevalence rate rose from 1.8% in 1988 to 5.8% in 2001. Since 1991 the Federal Ministry of Health has carried a National HIV/Syphilis Sentinel zero prevalence survey every 2 years. The most recent survey was in 2009 and it was estimated that there were 2.98 million people are living with HIV/AIDS in Nigeria with a total AIDS death of 192,000. Despite national prevalence of 4.6%, there are several variations by state and local government area. At the zonal level, prevalence is lowest in the South West (2.0%) and highest in the South-South (7.0%). Overall, 13 of Nigeria 36 states have a prevalence rate of over 5%^23^. These figures give support to the claim that there are explosive, localized epidemics in some states. Nigeria STD/HIV control estimated that over 60% of new HIV infections are in the 15–25 years old age group and this has been attributed to lack of knowledge regarding HIV/AIDS^24^.

The World Health Organization asserted that knowledge is a prerequisite for any action. Knowledge people have about any disease condition determines what they do about it^25^, ^26^. Adequate HIV/AIDS education provides an individual with enough information to be able to make balanced moral decisions about sexual behaviour. Kilander asserted that knowledge gives meaning to emotional attitude and strengthens it^27^. By implication ones knowledge about any object might determine the person's attitude towards the given object. According to WHO, there are 34.4 million people living with HIV/AIDS world wide^28^. Out of which 66% of them are in sub-Saharan African and 75% of deaths from AIDS are from sub-Saharan Africa^5^.

## The problem

Stigma and discrimination have long been identified as major obstacles that keep PLWHA from accessing prevention, treatment, and care services. According to the 2003 Ghana DHS, only 15% of men and 8% of women were found to have accepting attitudes towards people with HIV. In Guinea, Nigeria, and Senegal, accepting attitudes towards people with HIV were even lower, with fewer than 10% of men and fewer than 5% of women expressing accepting attitudes towards PLWHA. Stigma often leads to discrimination and other violations of human rights that affect the well-being of PLWHA^29^.

Studies^30^–^37^ in some parts of the globe have shown that due to stigmatization of PLWHA, cases that are not yet known hide the problem and continue to spread the scourge unnoticed. It is necessary to ensure that teachers have basic correct knowledge about HIV/AIDS^38^. This basic knowledge is a key factor in helping the teachers to overcome their fears, ignorance and prejudices and also to reduce the spread of HIV/AIDS when they exhibit positive attitude towards PLWHA. However, lack of knowledge about HIV/AIDS is one of the possible barriers to HIV/AIDS prevention^39^. It is therefore essential to identify the HIV/AIDS knowledge and attitude towards PLWHA among primary school teachers in Ebonyi State where it has not been established whether a study of this nature has been conducted.

Thus the purpose of the study was to determine HIV/AIDS knowledge and attitude towards PLWHA among primary school teachers in Ebonyi State. Specifically, the study was designed to:

1. determine HIV/AIDS knowledge and attitude towards people living with HIV/AIDS (PLWHA) among primary school teachers in Ebonyi State.
2. test if HIV/AIDS knowledge and attitude towards people living with HIV/AIDS (PLWHA) differ between male and female teachers.

Data generated in this study will likely be useful to the Academic Planning Units of Universities and Colleges of Education to enable them include HIV/AIDS education in the curriculum for training teachers, the discipline pursued by the intending teacher notwithstanding. However, Health Education curriculum planners will find the data very useful in order to include family life and HIV/AIDS education topics that would enable intending health education teachers have a priori knowledge of HIV/AIDS. It has been suggested that a good knowledge of HIV/AIDS would make people avoid discrimination against PLWHA^40^. Data emanating from the study could also serve as baseline information on the knowledge of HIV/AIDS and attitude towards PLWHA among primary school teachers in Ebonyi State.

## Methods

Between March and June 2016, a cross sectional survey was carried out among 400 primary school teachers of both genders drawn from 40 primary schools in Ebonyi State. The primary schools were selected from two (Ebonyi North and Ebonyi South) out of three education zones in Ebonyi State. In each school, 5 male and 5 female teachers were randomly selected using the stochastic technique.

The cross sectional survey used a researchers-developed questionnaire, called HIV/AIDS knowledge and attitude towards people living with HIV/AIDS questionnaire (HKAPLWHAQ), which consisted of 21 items arranged in three sections A, B, and C. section A contained one question about the gender of the respondents. Section B, comprised 10 ‘true or false’ (e.g., many people who are infected with HIV can look and feel healthy, T/F) knowledge of HIV/AIDS. Section C, contained 10 items (e.g., I wouldn't mind having a pupil with HIV in my classroom) meant to elicit information on attitude of the respondents towards PLWHA.

Three experts in Health Education from one institution of higher learning in Enugu State were used for validating the HKAPLWHAQ. Thirty primary school teachers of both genders in Ebonyi Central Education zone, not included in the study were used for test of reliability. The data yielded a reliability of r = 0.88 on the HKAPLWHAQ. A further reliability computation of each duster (HIV/AIDS knowledge r = 0.81; attitude towards PLWHA r = 0.84) of the HKPLWHAQ was done. The reliability coefficients were high enough considering Ogbazi and Okpala's criteria of 0.60 acceptable for good instruments^41^.

The permission to use the teachers for the research from the head teacher in each primary school included in the study was obtained before data collection. A consent note with the explanation of the research purpose, method of response and assurance of anonymity was attached to each copy of the HKAPLWHAQ. Because of the knowledge questions, copies of the HKAPLWHAQ were administered on the respondents in their respective classrooms during break period. The researchers stayed with the respondents while they were completing the questionnaire copies. This method was adopted in order to avoid any possible interaction during the process of responding to the questionnaire. The respondents were allowed 25 minutes to respond to the copies of the HKAPLWHAQ and return them immediately.

The completed copies of the HKAPLWHAQ were examined for completeness of responses and copies that had incomplete responses were discarded. Out of 400 copies of the HKAPLWHAQ administered; 394 (male 199, female 195) representing about 98.5% return rate, were used for analysis. Percentages were used to describe the respondents' HIV/AIDS knowledge. In describing the respondents' HIV/AIDS knowledge, a proportion of less than 20% correct responses was considered “very low” level of knowledge 21–39%, ‘low’; 40–59% ‘moderate’; 60–80% ‘high’, and above 80%; ‘very high’^42^, ^43^. Chi-sqare test was run to examine the differences in HIV/AIDS knowledge between male and female primary school teachers. Mean scores were used to describe the attitude of the respondents towards PLWHA. In describing the respondents' attitude, a mean score of 2.50 and higher was adjudged positive attitude and a mean score of less than 2.50 was adjudged negative attitude towards PLWHA. Standard deviations were used to examine how the responses given by the respondents varied. T-test was run to examine the differences in attitude of the respondents towards PLWHA in relation to gender. An alpha level of 0.05 was set for both sets of chi-square tests and t-tests tests. All data analyses were done with IBM SPSS version 23.0 for windows.

## Results

Percentages of correct responses to the HIV/AIDS questions and results of χ2-tests are listed in Table 1. The respondents possess moderate to very high level of knowledge in overall and most aspects of HIV/AIDS. However, the respondents possess low level of knowledge with regard to curability of AIDS and semen as a vehicle of transmission. When male respondents are compared with the females, while males are superior to females in six aspects of HIV/AIDS (e.g., curability of AIDS, semen as a vehicle of transmission, mother to child transmission, reduction of chances of becoming infected with HIV by use of latex condom, being careful to have sexual intercourse only with healthy-looking partners won't become infected with HIV and being infected with HIV and not knowing) and in overall knowledge; females demonstrate better knowledge in the other four aspects (e.g., infected persons looking healthy, transmission through blood, transmission of HIV through mosquito bite and becoming infected by donating blood) than males. Chi-square (χ2) tests indicate there is no significant difference in the possession of knowledge between male and female respondents in most aspects of HIV/AIDS and overall expect in three aspects where a significant difference exists.

**Table 1:** Percentages and Chi-square tests results of knowledge of HIV/AIDS between male and female primary school teachers (N = 394)

Knowledge Statement	% of correct responses				
	Total	Male	Female		
	%	%	%	χ^2^- value	p-value
(**T**) Many people who are infected with HIV can look and feel healthy.	70.1	63.3	76.9	8.691*	0.003
(**F**) AIDS can be cured.	36.3	19.8	16.5	1.464	0.226
(**T**) Males who are infected with HIV can give it to another person through their semen.	23.6	39.2	20.0	2.781	0.095
(**T**) People who are infected with HIV can give it to another person through their blood.	75.6	73.4	77.9	1.122	0.289
(**T**) A mother can pass HIV to her unborn child.	54.8	56.8	52.8	0.625	0.429
(**T**) People can reduce their chances of becoming infected with HIV by using a latex condom during sexual intercourse.	41.4	46.2	36.4	3.916*	0.048
(**F**) A person can become infected with HIV by being bitten by an insect such as a mosquito.	49.7	46.7	52.8	1.460	0.227
(**F**) A person can become infected with HIV by donating (giving) blood.	67.5	67.3	67.7	0.006	0.938
(**F**) People who are careful to have sexual intercourse only with healthy-looking partners won't become infected with HIV.	76.9	82.9	70.8	8.179*	0.004
(**T**) People can be infected with HIV and not know they have it.	77.7	77.4	72.9	0.018	0.893

**Overall**	**57.4**	**58.0**	**56.7**	**0.030**	**0.843**

**T** = True, **F** = False

* Significant at *p* < .05

Means, standard deviations and t-tests results of attitude of the respondents towards PLWHA are listed in Table 2. The results show that the respondents exhibit, overall, a positive attitude (3.09 ± 0.98) towards PL-WHA and in all attitude statements. When male and female respondents are compared, females (= 3.16, SD 0.91) show a more favourable attitude towards PLWHA than males (= 3.00, SD 1.01) do. However, significant differences exist in five out of the ten attitude items towards PLWHA measures; but no significant difference exists in the overall attitude towards PLWHA between male and female respondents.

**Table 2:** Means, standard deviations and t-tests results of attitude towards PLWHA questions between male and female primary school teachers

Attitude Statement	Mean responses				
	Total	Male	Female		
	*x̅* (SD)	*x̅* (SD)	*x̅* (SD)	t -value	p-value
I wouldn't mind having a pupil with HIV in my classroom.	3.10 (1.17)	3.12 (1.14)	3.08 (1.21)	0.264	0.712
A pupil who is infected with HIV should be able to eat lunch with other students.	2.98 (1.16)	2.89 (1.19)	3.07 (1.12)	1.567	0.118
I wouldn't avoid a pupil whose family member had AIDS.	3.03 (1.15)	2.91 (1.19)	3.14 (1.10)	1.935	0.054
I would work with another teacher who was infected with HIV.	3.01 (1.17)	3.00 (1.19)	3.03 (1.15)	0.217	0.828
Pupils infected with HIV shouldn't be separated from other students.	3.08 (1.12)	3.02 (1.14)	3.14 (1.10)	1.047	0.296
Pupils who are infected with HIV should play sports with other students.	3.11 (1.14)	3.00 (1.15)	3.22 (1.11)	1.935	0.054
I would feel comfortable about giving individual help to a pupil infected with HIV.	3.19 (1.16)	3.06 (1.22)	3.33 (1.08)	2.397*	0.017
People who have AIDS should be allowed to work in places that handle food.	3.17 (1.14)	3.04 (1.19)	3.03 (1.07)	2.248*	0.025
If I thought a fellow teacher was infected with HIV, I wouldn't be afraid to shake hands with that teacher.	3.17 (1.17)	3.02 (1.25)	3.32 (1.07)	2.587*	0.010
I would feel comfortable hugging a friend who has AIDS.	3.06 (1.21)	2.92 (1.29)	3.21 (1.10)	2.317*	0.021

**Overall**	**3.09 (0.98)**	**3.00 (1.01)**	**3.16 (0.91)**	**1.379**	**0.169**

Figures in brackets are standard deviations

* Significant at *p* < 0.05

## Discussion

The present study determined HIV/AIDS knowledge and attitude towards PLWHA among primary school teachers in Ebonyi State. Results of the study showed that; overall, the respondents had moderate level of knowledge about HIV/AIDS. However, misconceptions still existed, especially with regard to curability of AIDS and transmission of HIV from males who are infected to another person through their semen. Alarming gaps in knowledge about transmission and curability could put the teachers and young students they teach at risk of contracting HIV. Tolerable attitudes towards people living with HIV were prevalent. Present results suggest that a universal health education programme, focusing on family life and HIV/AIDS education, in tertiary institutions in Nigeria may be necessary to reduce the discrepancy knowledge regarding curability of AIDS.

The respondents having a moderate level of knowledge of overall HIV/AIDS was not plausible since, according to Silverman knowledge people have about any disease condition determines what they do about the condition^26^. Recent studies^44^–^48^ in both developed and developing countries had revealed findings that are at variance with those of the present study. The results also showed that overall there was no significant difference between male and female respondents in the level of HIV/AIDS they possessed. That no significant difference existed between the knowledge possessed by male and female teachers may not attract any question because naturally a teacher is a teacher, no matter the gender. Previous studies^49^,^50^ had found gender differences in HIV/AIDS knowledge among the students they studied. The variation in the status of respondents used in the present study my have accounted for the inconsistency in the findings of the present study with those of previous ones.

Results in Table 2 showed that the teachers had a positive attitude towards PLWHA, stating in some instances that, ‘*I wouldn't mind having a pupil with HIV in my classroom, a pupil who is infected with HIV should be able to eat lunch with other students, I would work with another teacher who was infected with HIV, I would feel comfortable about giving individual help to a pupil infected with HIV, and if I thought a fellow teacher was infected with HIV, I wouldn't be afraid to shake hands with that teacher*’, among other positive statements. Disagreement about the statement above by respondents would have suggested a feeling of discrimination towards PLWHA with the attendant implication of HIV/AIDS-related stigma^51^–^54^. The positive attitude exhibited by the teachers seems to corroborate the knowledge they possessed about HIV/AIDS. This favourable attitude towards PLWHA was not observed among a group of people^55^–^60^. However, the positive attitude found in the present study corroborated the findings of a previous study^61^.

When the attitude expressed by female teachers was compared with that expressed by male teachers, female teachers showed a more favourable attitude than male teachers but the difference was not significant (p > 0.05). However, previous studies^62^,^63^ had observed significant differences in the attitude towards PLWHA among their study sample.

## Conclusion and recommendation

HIV/AIDS knowledge and attitude towards PLWHA is important in the prevention of the scourge. This scenario is especially important among teachers who are charged with the responsibility of educating the youth. Misconceptions still existed among the teachers studied, especially with regard to curability of AIDS and transmission of HIV from males who are infected to another person through their semen. However, the teachers demonstrated a moderate level of knowledge concerning HIV/AIDS. Alarming gaps in knowledge about transmission and curability could put the teachers and young pupils they teach at risk of contracting HIV. Tolerable attitudes towards people living with HIV were prevalent.

The results of the study may not be used in making a sweeping conclusion concerning other groups of teachers in Nigeria and elsewhere; who may differ to a large extent in social and economic conditions. The teachers studied represent an important group of the Nigerian population and information generated will be useful in the planning of future sexually transmitted infections and other health-related programmes in tertiary institutions in Nigeria and may be other sub-Saharan Africa countries. Since teachers play a key role in teaching young people about HIV/AIDS, the present study suggests inclusion of a universal health education programme, focusing on HIV/AIDS education, in education institutions that train teachers in Nigeria may be necessary to reduce the discrepancy in knowledge regarding curability of AIDS and any undesirable attitude towards PLWHA that may arise among teachers.
